# The Treatment of a Cold

**Published:** 1883-10

**Authors:** 


### EDITORIAL, ETC.

---

THE TREATMENT OF A COLD.—" The London Monthly *Magazine* reports Dr. Graham as saying that it is not a correct practice, after a cold is caught, to make the room a person sits in much warmer than usual, to increase the quantity of bed-clothes, wrap up in flannel, and drink a large quantity of hot tea, gruel or other slops, because it will invariably increase the feverishness, and, in the majority of instances, prolong rather than lessen the duration of the cold. It is well known that confining innoculated persons in warm rooms will make their small-pox more violent by augmenting the general heat and fever ; and it is for the same reason that a similar practice in the present complaint is attended with analygous results, a cold being in reality a slight fever. In some parts of England, among the lower class of people, a large glass of cold spring water, taken on going to bed, is found to be a successful remedy, and in fact, many medical practitioners recommend a reduced atmosphere and frequent draughts of cold fluid as the most efficacious remedy for a recent cold, particularly when the patients habit is full and plethoric.

Dr. Graham further says : It is generally supposed that it is the exposure to a cold or wet atmosphere which produces the effect called cold, whereas it is returning to a warm temperature after exposure which is the real cause of the evil. When a person in the cold weather goes into the open air, every time he draws in his breath the cold air passes through his nostrils and windpipe into his lungs, and consequently diminishes the heat of those parts. As long as the person continues in the cold air he feels no bad effects from it; but as soon as he returns home he approaches the fire to warm himself, and very often takes some warm and comfortable drink to keep out the cold, as it is said. The inevitable consequence is that he will find he has taken cold. He feels a shivering which makes him draw nearer the fire, but all to no purpose; the more he tries to heat himself the more he chills. All the mischief is here caused by the violent action of the heat.

To avoid this when you come out of a very cold atmosphere, you should not at first go into a room that has a fire in it, or if you cannot avoid that, you should keep for a considerable time at as great a distance as possible, and, above all, refrain from taking warm or strong liquors when you are cold. This rule is founded on the same principle as the treatment of any part of the body when frost-bitten. If it were brought to the fire it would soon mortify, whereas if rubbed with snow no bad consequences follow from it. Hence, if the following rule were strictly observed—when the whole body, or any part of it is chilled, bring it to its natural feeling and warmth by degrees—the frequent colds we experience in winter would in a great measure be prevented.

